# The Expanding Family of Natural Anion Channelrhodopsins Reveals Large Variations in Kinetics, Conductance, and Spectral Sensitivity

**DOI:** 10.1038/srep43358

**Published:** 2017-03-03

**Authors:** Elena G. Govorunova, Oleg A. Sineshchekov, Elsa M. Rodarte, Roger Janz, Olivier Morelle, Michael Melkonian, Gane K.-S. Wong, John L. Spudich

**Affiliations:** 1 Center for Membrane Biology, Department of Biochemistry & Molecular Biology, The University of Texas Health Science Center at Houston, McGovern Medical School, Houston, Texas, USA; 2 Department of Neurobiology & Anatomy, The University of Texas Health Science Center at Houston, McGovern Medical School, Houston, Texas, USA; 3 Institute of Botany, Cologne Biocenter, University of Cologne, Cologne, Germany; 4 Departments of Biological Sciences and of Medicine, University of Alberta, Edmonton, Alberta, Canada; 5 BGI-Shenzhen, Shenzhen, China

## Abstract

Natural anion channelrhodopsins (ACRs) discovered in the cryptophyte alga *Guillardia theta* generate large hyperpolarizing currents at membrane potentials above the Nernst equilibrium potential for Cl^−^ and thus can be used as efficient inhibitory tools for optogenetics. We have identified and characterized new ACR homologs in different cryptophyte species, showing that all of them are anion-selective, and thus expanded this protein family to 20 functionally confirmed members. Sequence comparison of natural ACRs and engineered Cl^−^-conducting mutants of cation channelrhodopsins (CCRs) showed radical differences in their anion selectivity filters. In particular, the Glu90 residue in channelrhodopsin 2, which needed to be mutated to a neutral or alkaline residue to confer anion selectivity to CCRs, is nevertheless conserved in all of the ACRs identified. The new ACRs showed a large variation of the amplitude, kinetics, and spectral sensitivity of their photocurrents. A notable variant, designated “ZipACR”, is particularly promising for inhibitory optogenetics because of its combination of larger current amplitudes than those of previously reported ACRs and an unprecedentedly fast conductance cycle (current half-decay time 2–4 ms depending on voltage). ZipACR expressed in cultured mouse hippocampal neurons enabled precise photoinhibition of individual spikes in trains of up to 50 Hz frequency.

Versatile control of the membrane potential in optogenetic studies requires both depolarizing and hyperpolarizing tools. Cation channelrhodopsins from green (chlorophyte) algae (CCRs) are widely used to depolarize the membrane and stimulate neuronal activity^1^. Hyperpolarization of the membrane and inhibition of neuronal firing by light was first achieved with rhodopsin ion pumps^2^,^3^. However, these molecules transport only one charge per absorbed photon, which intrinsically limits their efficiency. They have been successfully used to silence neurons in live animals, but have required high-density expression and intense light (10–20 mW/mm2^4^,^5^,^6^). To create better inhibitory tools, CCRs were engineered by mutagenesis to conduct Cl^−^ 7,8,9,10. The latest version of the fast mutants, iC++, permits inhibition of evoked neuronal spiking at higher injected currents than the Cl^−^ pump eNpHR3.0 (enhanced *Natronomonas pharaonis* halorhodopsin version 3.0)^10^. The light sensitivity of the Cl^−^-conducting CCR mutants was further increased by an additional mutation that extends the photocycle duration^7^,^8^. The slow mutants can be switched off with red light to improve temporal control of inhibition^10^, but this photochromicity does not eliminate the need for a long illumination to reach the maximal current amplitude.

Recently discovered natural anion channelrhodopsins (ACRs) from the cryptophyte alga *Guillardia theta* have fast photocycles and 10,000-fold higher sensitivity than previously used fast silencing tools^11^. ACR expression in excitable cells allowed specific and robust inhibition of several behavioral responses in live *Drosophila* at light intensities much lower than those required with eNpHR^12^. Much more efficient inhibition of action potentials by ACRs than by the proton pump archaeorhodopsin-3 (Arch) at the unperturbed transmembrane Cl^−^ gradient has been demonstrated by extracellular recording from cultured rat ventricular cardiomyocytes^13^. A homologous protein (*Psu*ACR1) from another cryptophyte, *Proteomonas sulcata*, also showed anion selectivity^14^,^15^, indicating that ACRs are not confined to a single species.

Here we report the identification of new ACR homologs in the transcriptomes of diverse cryptophyte species and electrophysiological analysis of those that produced photocurrents when expressed in cultured mammalian cells. All exhibited anion selectivity, as did the first discovered *G. theta* ACRs (*Gt*ACRs). Sequence alignment of 20 functionally confirmed ACRs shows that the naturally evolved anion conductance has a different structural basis than that created in engineered Cl^−^-conducting mutants of CCRs. The new natural ACRs demonstrated large variation of their spectral and kinetic properties.

Earlier reported ACRs are appealing either for their large (*Gt*ACRs) or relatively fast-decaying (*Psu*ACR1) photocurrents. One of the new ACRs, named *Psu*ACR_973 or “ZipACR” for its fast kinetics, excels in both of these characteristics, being superior to *Psu*ACR1 in its current decay rate and to *Gt*ACRs in its current amplitude. In addition, its spectral sensitivity is more red-shifted than that of *Gt*ACR2 previously used to suppress neuronal firing. ZipACR expression in cultured mouse hippocampal neurons enabled selective inhibition of individual spikes in neurons firing at 50 Hz, the upper frequency limit for regular spiking in this neuronal type^16^.

## Results

### Sequences of the new ACR homologs

To identify transcripts encoding proteins homologous to earlier known ACRs from *G. theta*^11^ and *P. sulcata*^14^ we searched algal transcriptomes generated by the Marine Microbial Eukaryote Transcriptome Sequencing Project (MMETSP)^17^ and 1,000 Plants Project (1KP)^18^. Of all algal groups, ACR homologs were found only in cryptophytes; Supplementary Table S1 contains a list of analyzed cryptophyte species, their habitats and the output of the search. No ACR homologs were found in freshwater species, but only a small number of such have been analyzed. We synthesized mammalian codon-adapted versions of the seven-transmembrane-helix domains of ACR homologs, the protein alignment of which is shown in Supplementary Fig. S1. Usually, the first letters of the genus and species names are used in abbreviated rhodopsin names, e.g. *Gt* for *G. theta*, and we followed this convention when possible. However, because one *Geminigera* strain and two *Rhodomonas* strains from which the new sequences were derived were not classified at the species level, we used italicized numbers instead of the species names in protein abbreviations, i.e. *G1, R1* and *R2*. Two transcripts originated from a cryptophyte strain that has not yet been classified at the genus level, so we included *C1* in the genus-species position in the corresponding protein names.

**Figure 1:**
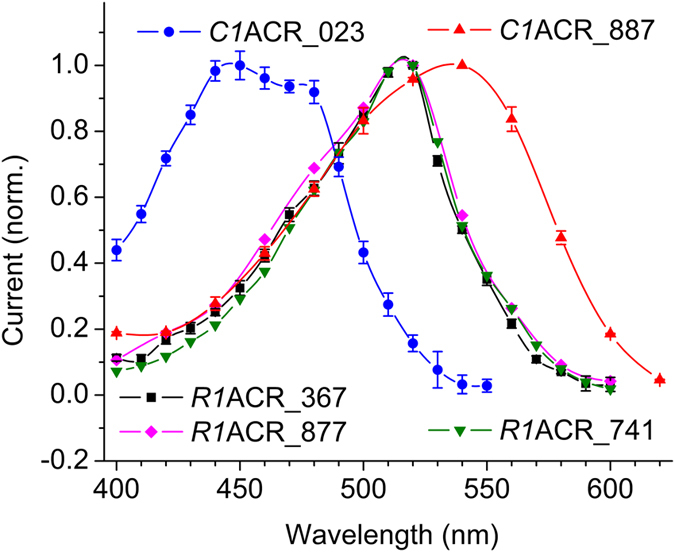
Action spectra of photocurrents generated in HEK293 cells. An example of a spectrally shifted ACR pair from a single cryptophyte species (*C1*ACR_023 and *C1*ACR_887), and an example of three spectrally matching ACRs from another species (*R1*ACR_367, *R1*ACR_877 and *R1*ACR_741). The initial slopes of photocurrents were measured in the linear range of the intensity dependence, corrected for the quantum density, and normalized to the maximal value obtained for each protein (for more detail see Methods). The data points are the mean values ± sem (n = 4–6 measurements in 3–4 cells). The spectra of other proteins were measured in a similar way and the resultant spectral maxima are listed in Table 1.

The transcripts were fused in frame with the C-terminal enhanced yellow fluorescent protein (EYFP) tag, and human embryonic kidney (HEK293) cells were transfected with the resultant constructs. Seventeen constructs generated transmembrane currents in response to illumination (Supplementary Fig. S2); two constructs that did not generate currents were excluded from further analysis. The spectral sensitivity was determined as described previously^19^. In most chlorophyte (green) algae only two channelrhodopsin genes have been found^20^, and a historical convention is to assign the number “1” to the more red-shifted variant from the same species, and number “2”, to the more blue-shifted one^21^,^22^. Such spectrally shifted pairs also exist among ACR homologs derived from the same source organism (Fig. 1, *C1*ACR_023 and *C1*ACR_887). However, in some cryptophyte species the number of ACR paralogs was larger than two, and the difference between the spectral maxima of their photocurrents was very small, if any (Fig. 1, the three *R1*ACR paralogs). Therefore, we distinguished them by including the last three digits of the transcript name in the abbreviated protein names. GenBank accession numbers, source organisms, transcript names, the wavelength of the maximal sensitivity and protein name abbreviations are listed in Table 1.

### All of the new ACR homologs are natural chloride-conducting channelrhodopsins

**Figure 2:**
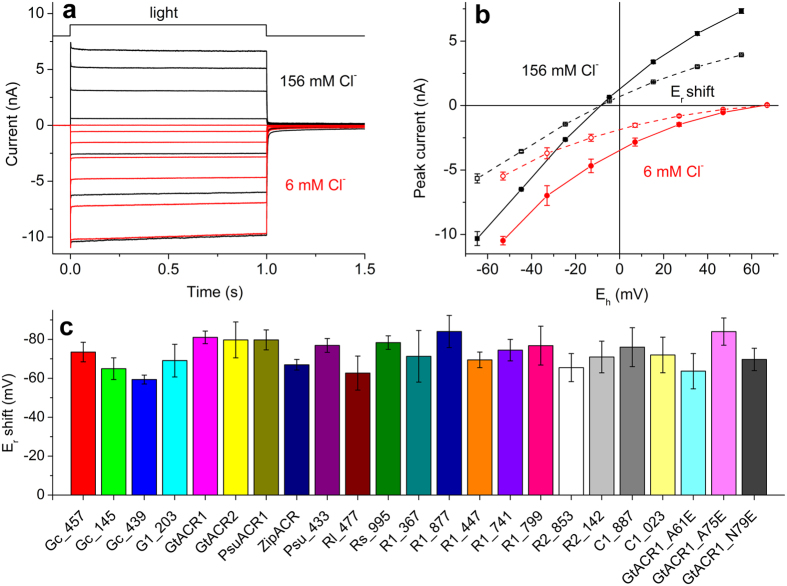
All functional ACR homologs conduct Cl^−^. (**a**) A representative series of photocurrent traces recorded in response to a 1-s light pulse of the saturating intensity from *Psu*ACR*_*973 (ZipACR) expressed in HEK293 cells in the standard bath (black lines) and upon partial replacement of Cl^−^ with Asp^−^ in the bath (red lines). The holding voltage (E_h_) was changed in 20-mV steps from −60 mV at the amplifier output (bottom trace). (**b**) The current-voltage relationships of peak (filled symbols) and stationary (empty symbols) photocurrents determined from current traces as shown in panel a. The data points are the mean values ± sem (n = 3 measurements in the same typical cell). The data were corrected for liquid junction potentials. (**c**) The E_r_ shifts (E_r_ in the standard bath minus E_r_ in the Asp bath) measured for the currents averaged over the entire 1-s illumination period for all tested ACR homologs and *Gt*ACR1 mutants. The data are mean values ± sd (n = 3–5 cells). The data for the earlier studied ACRs are included for comparison.

To test whether the new ACR homologs indeed conduct anions we measured the current-voltage relationships and determined the shift of the reversal potentials (E_r_) upon partial replacement of Cl^−^ in the bath with non-permeable Asp^−^, as was done earlier for *Gt*ACRs and *Psu*ACR1^11^,^14^. Representative series of currents traces recorded from *Psu*ACR*_*973 (nicknamed ZipACR based on its robust currents, rapid kinetics, and function in neuron silencing shown below) in the standard bath (black lines) and after replacement (red lines) are shown in Fig. 2a, and the corresponding current-voltage relationships (IE curves) for the peak and stationary currents are shown in Fig. 2b. In contrast to some CCRs^23^, ionic selectivity of natural ACRs does not change during illumination (Fig. 2b). The difference between the E_r_ measured for the peak and stationary currents was <2 mV for all tested natural ACRs. Therefore, E_r_ shifts were calculated for the currents averaged over the entire 1-s illumination period to improve the accuracy of measurements. The results showed that all tested ACR homologs conduct anions (Fig. 2c).

### Structural determinants of anion selectivity in natural channelrhodopsins

**Figure 3:**
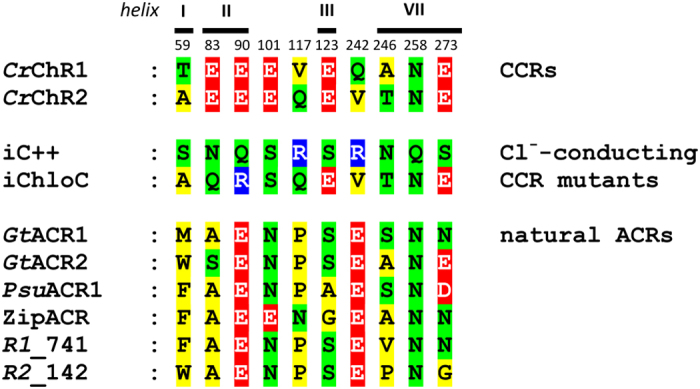
Comparison of the mutations introduced to confer Cl^−^ selectivity to CCRs with the corresponding residues in natural ACRs. The color code is: red, negatively charged residues; blue, positively charged residues; green, polar residues; yellow, non-polar residues. The numbers on top show the residue numbers according to the *Cr*ChR2 sequence.

With a set of now 20 functionally confirmed ACRs we performed protein sequence analysis to compare natural cryptophyte ACRs with Cl^−^-conducting mutants engineered from chlorophyte CCRs. The first two lines in Fig. 3 show amino acid residues of CCR sequences. The third and fourth lines are the mutations introduced to confer Cl^*−*^ selectivity to the latest versions of Cl^*−*^-conductive mutants of CCRs, iC++^10^ and iChloC^9^, respectively. The remaining lines show the corresponding residues in six of the confirmed natural ACRs (for the residues in the remaining 14 sequences see Supplementary Fig. S1).

The most revealing difference between the artificial and natural Cl^*−*^-conductive proteins is the position corresponding to Glu90 in *Cr*ChR2: it needed to be replaced with a neutral or positively charged residue to convert a CCR into a Cl^*−*^ channel, whereas it is present in all natural ACRs. Moreover, replacement of the Glu90 homolog Glu68 in *Gt*ACR1 with a neutral (Gln) or alkaline (Lys) residue does not alter its anion selectivity, but rather strongly influences the ACR’s gating properties^24^, both of these effects in stark contrast to the corresponding mutations in *Cr*ChR2^7^. Furthermore, out of two positions at which positive charges were introduced in iC++, one (Gln-117 in *Cr*ChR2) is occupied with a neutral residue, and another (Val242 in *Cr*ChR2), with a negative charge in all confirmed natural ACRs (Fig. 3 and Supplementary Fig. S1). These findings indicate that the anion conduction pathways in natural and artificial ACRs are very different and the conserved Glu90 residue in helix 2 has diverged to serve different roles in the CCR and ACR mechanisms.

In addition to the aforementioned Glu90, most CCRs contain four other Glu residues in the 2^nd^ helix and the loop between helices 2 and 3 (Glu82, Glu83, Glu90, Glu97 and Glu101 in *Cr*ChR2). It has been proposed that the absence of Glu residues in the positions corresponding to Glu83, Glu97 and Glu101 contributes to the formation of an anion-conducting pore in natural ACRs^10^. Indeed, in nearly all confirmed natural ACRs non-carboxylic residues are found in these three positions (Supplementary Fig. S1). However in the highly conductive ZipACR (Fig. 2), Glu101 is conserved (Supplementary Fig. S1), which demonstrates that the presence of a non-carboxylate residue at least in this position is not absolutely necessary for anion conductance.

To further test the hypothesis that the absence of Glu83, Glu97 and Glu101 determines anion conductance of natural ACRs, we introduced these glutamates in the corresponding positions in *Gt*ACR1. Glu83 and Glu97 of *Cr*ChR2 are located in the 2^nd^ transmembrane helix, which was confirmed by the high-resolution crystal structure of a hybrid channelrhodopsin^25^. The corresponding residues in the predicted 2^nd^ helix of *Gt*ACR1 are Ala61 and Ala75 (Supplementary Fig. S3). Glu101 of *Cr*ChR2 is located in the 2^nd^ loop; therefore to determine its counterpart in *Gt*ACR1 is more difficult. Gln79 in *Gt*ACR1 is separated by 3 residues from Ala75, as is Glu101 from Glu97 in *Cr*ChR2. We generated the A61E, A75E and N79E mutants of *Gt*ACR1, expressed them in HEK293 cells and measured their IE curves in the standard buffer and upon partial replacement of Cl^−^ with Asp^−^ in the bath, as we did for natural ACR homologs. The shifts of the E_r_ upon such replacement determined for each of these mutants were almost as large as those of natural ACRs (Fig. 2c). Therefore, we conclude that the presence of individual conserved residues in the 2^nd^ helix is not sufficient to predict anion or cation selectivity of channelrhodopsins. This conclusion is corroborated by an earlier observation that *Mesostigma viride* channelrhodopsin 1 (*Mv*ChR1) in which Glu83 and Glu97 homologs are also replaced with neutral residues as in ACRs is in fact a CCR with one of the highest Na^+^/H^+^ permeability ratios among CCRs^19^. However, the A61E and A75E mutations of *Gt*ACR1 strongly decreased the current amplitude. In the standard bath (pH 7.4) at −60 mV the peak current of the A61E mutant was 48 ± 9 pA (n = 11 cells) and that of the A75E mutant, 109 ± 21 pA (n = 14 cells). On the other hand, the N79E mutation did not affect the peak current amplitude (6.0 ± 4.3 nA, n = 3 cells).

Cl^−^-conducting CCR mutants in which the residue corresponding to Glu83 of *Cr*ChR2 was retained generated larger photocurrents at low pH than at neutral pH^7^,^8^. The photocurrent of the A61E mutant in which a Glu residue was introduced in the corresponding position in *Gt*ACR1 did not increase at pH 5.4 (51 ± 19 pA; n = 8 cells) as compared to that at pH 7.4. This observation further confirmed the difference in anion conductance mechanisms in engineered CCR mutants and natural ACRs.

### Functional Diversity in the ACR family

**Figure 4:**
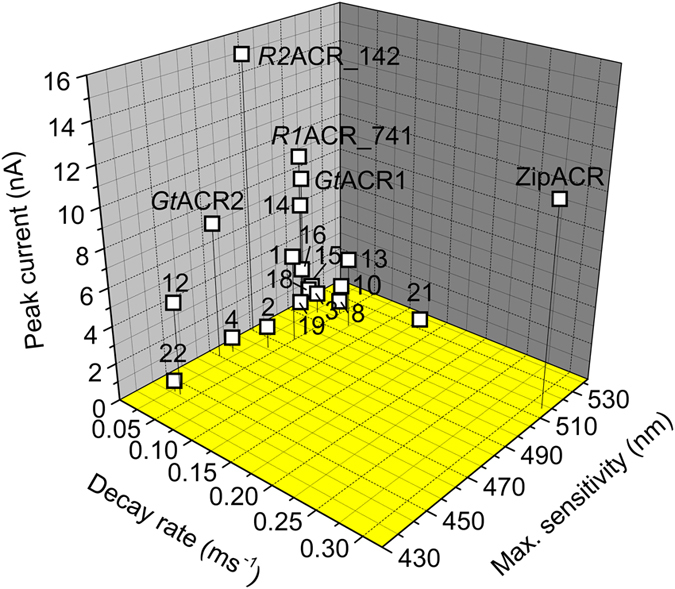
ACR functional diversity. The peak current amplitude in response to a 1-s light pulse of the saturating intensity, decay rate (measured as the reciprocal of the time of the 50% decrease in the current amplitude after the light-off) and wavelength of maximal sensitivity (determined by continuous approximation of experimental data points as shown in Fig. 1) of cryptophyte ACR homologs. The amplitude and decay rate data were obtained at −60 mV holding potential in standard solutions (see Methods). The data points are the mean values; for sem values and the number of sampled cells see Supplementary Table S2. The numbers next to the data points correspond to the protein numbers in Table 1. The data for the previously known *Gt*ACR1, *Gt*ACR2 and *Psu*ACR1 are included for comparison.

Photocurrents generated by new ACRs greatly differed in amplitude, kinetics and spectral sensitivity, demonstrating diversity of this protein family (Fig. 4 and Supplementary Fig. S2). The range of the wavelengths of the ACR spectral maxima extended from 445 (*C1*ACR_023) to 535 (*C1*ACR_887) nm. The degree of current inactivation during 1-s continuous illumination varied between 12.5% (*R1*ACR_877) and 83.6% (*R2* ACR_853). *Psu*ACR_973, *R1*ACR_741 and *R2*ACR_142 generated currents larger than those produced by the previously known ACRs. *Psu*ACR_973 also exhibited the by far fastest current decay among all so far identified ACRs (~2.2 ms current half-decay time at zero voltage; Supplementary Fig. S4 shows the voltage dependence of the current half-decay time), the main reason we nicknamed it “ZipACR”. The dependence of ZipACR photocurrent on the light intensity is shown in Supplementary Fig. S4.

Protein sequence alignment revealed that ZipACR differs from the majority of functionally confirmed ACRs in the positions 101, 117 and 123 (according to *Cr*ChR2 numbering). The corresponding residues in ZipACR are Glu96, Asn108 and Gly114, whereas the majority of ACRs contain, respectively, Asn, Pro and Ser in these positions. We generated the E96N, N108P and G114S mutants of ZipACR and measured their photocurrents. No differences were found in the current decay rate between the mutants and the wild type (Supplementary Fig. S5), which showed that the mutated residues do not determine the fast decay kinetics of ZipACR.

### ZipACR allows optical inhibition of individual neuronal spikes at unprecedentedly high frequencies

**Figure 5:**
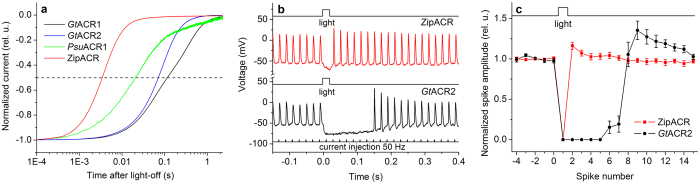
ZipACR is a fast inhibitory tool for optogenetics. (**a**) The decay of photocurrents generated by ACRs indicated in the figure legend in HEK293 cells after a 1-s pulse of continuous light of the saturating intensity at the wavelength of the peak absorption of the respective pigment. The traces were normalized at the stationary level measured near the end of the illumination period. (**b**) Photoinduced spike suppression in cultured mouse hippocampal neurons expressing ZipACR (red line) or *Gt*ACR2 (black line). Neurons were stimulated by injection of 1-ms current pulses at 50 Hz (schematically shown below). The wavelength of the 20-ms light pulses of the saturating intensity (shown on top of each trace) was 520 nm for ZipACR and 470 nm for *Gt*ACR2. (**c**) Photoinduced changes in the normalized spike amplitude measured as shown in panel b. The data points are the mean values ± sem (n = 7 cells). The amplitude was normalized to the mean amplitude of the last five spikes before switching on the light.

*Gt*ACR2, the fastest of the two *G. theta* ACRs, could be used in neurons to suppress individual spikes at firing frequencies up to 25 Hz^11^. However, its current decay is too slow for selective deletion of individual spikes in trains of higher frequency that may be required in certain optogenetic experiments^26^. Photocurrents from ZipACR exhibited ~21 times faster decay than those from *Gt*ACR2 in HEK293 cells, their amplitude was ~40% larger, and the wavelength of the maximal sensitivity was 45 nm red-shifted from that of *Gt*ACR2 (Fig. 4 and Supplementary Table S2). We expressed ZipACR in cultured mouse hippocampal neurons under the control of an ubiquitin promoter using lentiviral delivery and tested its performance as an inhibitory optogenetic tool compared to that of *Gt*ACR2. Robust expression of ZipACR in neurons was evident from the tag fluorescence, whereas neuronal morphology and physiology in the dark was unaffected (Supplementary Fig. S6). We stimulated the neurons by somatic current injection at 50 Hz, which is the maximal frequency of regular spiking in pyramidal neurons of the rodent hippocampus^16^. Delivery of a 20-ms light pulse of a saturating intensity resulted in suppression of an individual spike in ZipACR-expressing neurons (Fig. 5b, top trace), whereas in *Gt*ACR2-expressing cells up to seven successive spikes were suppressed (Fig. 5b, bottom trace). The ensemble data are shown in Fig. 5c. These properties make ZipACR a likely first choice for many optogenetic applications, especially in high-frequency neurons.

## Discussion

Here we present the results of screening ACR homologs identified in high-throughput plant/protist transcriptome sequencing projects^17^,^18^. We found that all transcripts that generated photocurrents upon expression in mammalian cells acted as light-gated anion channels. This result confirmed that cation or anion selectivity of channelrhodopsins can be accurately predicted from the protein sequence information or, in other words, that ACRs and CCRs form two structurally and functionally distinct protein families. When only three natural ACRs were known, testable prediction of structural determinants of their anion conductance was difficult. The new expanded set of 20 functional sequences will serve as a better guide towards this goal.

Two sequences closely related to functional ACRs were found to generate no measurable photocurrents upon heterologous expression. This was not unusual, because one of three ACR homologs in *G. theta* also generated no photocurrents despite its robust expression in animal cells^11^, and many sequences highly homologous to CCRs were non-electrogenic when expressed in HEK cells or neurons^27^,^28^. The most likely explanation is improper folding of these algal proteins in animal cells that does not influence fluorescence of the C-terminal tag but disrupts their channel function.

Comparison of the mutations introduced in CCRs to convert them into Cl^−^-conducting channels with the corresponding positions in natural ACRs revealed a difference rather than a “striking convergence” as stated elsewhere^10^,^26^. The most conspicuous feature is the universal conservation of Glu90 (according to *Cr*ChR2 numbering) in all tested ACRs, which was replaced with a positive charge in the ChloC and iChloC Cl^−^-conducting mutants^7^,^9^ and with a neutral residue in the iC1C2 and iC++ mutants^8^,^10^. According to our analysis of the laser-evoked photocurrents and spectral transitions of purified wild-type *Gt*ACR1 and its mutants, Glu68, which corresponds to Glu90 in *Cr*ChR2, likely serves as a negative counterion and proton acceptor at least at high and neutral pH^29^. A Resonance Raman spectroscopy study suggested that Glu68 exists in a neutral state in unphotolyzed *Gt*ACR1^30^, but notes that this residue may become transiently deprotonated during the photocycle as was earlier proposed for Glu169 in cation channelrhodopsin 1 from *Chlamydomonas augustae (Ca*ChR1)^31^.

In addition to the Glu90 homolog, all ACRs contain a Glu residue in the position of Val242 in *Cr*ChR2, in which a positive charge was introduced in the iC++ mutant^10^. Furthermore, we showed that although Glu residues corresponding to Glu83 and Glu101 of *Cr*ChR2, neutralized in Cl^−^-conducting mutants, are also neutral in most ACRs, introducing these glutamates in *Gt*ACR1 did not eliminate its anion selectivity.

Coupling between the chromophore photochemical reactions and channel opening in ACRs and CCRs appear to be very different. In CCRs the first phase of formation of an M-like intermediate with a deprotonated Schiff base occurs on the μs time scale, i.e. Schiff base proton transfer precedes channel opening^32^. In contrast, proton transfer accompanying M formation in the photocycle of ACRs is ~50 times slower than channel opening^15^,^29^.

In cation-conducting *Cr*ChR2 mutation of the Schiff base counterion (a homolog of Asp85 in bacteriorhodopsin) to a neutral residue resulted in profoundly accelerated kinetics of channel closing^33^. All so far confirmed ACRs contain a non-carboxylic residue in this position (Supplementary Fig. S1), but differ ~50 times in their current decay rate (Fig. 4 and Supplementary Table S2), indicating that it is regulated by other residues. Previously, we identified Glu68 (the homolog of Glu90 in *Cr*ChR2) in the middle of the 2^nd^ helix as required for the fast phase of channel closing in *Gt*ACR1^29^. This residue is universally conserved in ACRs, as is a homolog of Cys128 in *Cr*ChR2, mutation of which affects the slow phase of channel closing in *Gt*ACR1^29^. Photocurrents from all five ACRs found in *Rhodomonas* sp. strain CCMP768 exhibit slower decay as compared to ACRs from other cryptophytes, which probably reflects the cellular physiology of this alga.

Among new ACRs ZipACR appears to be most promising for optogenetic applications, because it exhibits much faster current decay comparable to that of rhodopsin ion pumps most frequently used as optogenetic inhibitors^3^,^34^. ZipACR current amplitude is even greater than that of the potent earlier studied *Gt*ACRs, and has long-wavelength sensitivity. In cultured mouse hippocampal neurons ZipACR outperformed *Gt*ACR2 for precise inhibition of individual spikes in 50-Hz trains, the highest frequency for sustained spiking in this neuronal type^16^. At 25-Hz stimulation suppression of individual spikes could also be achieved using *Gt*ACR2 and stimuli of lower intensity, but only at the expense of time resolution because a longer time was needed to reach sufficient hyperpolarization of the membrane^11^.

The upper limit of the firing frequency at which ZipACR can suppress individual spikes can be roughly estimated from the time constant of its current decay in neurons. The rate constant obtained by single exponential fit was 2.3 ± 0.4 ms (mean ± sem, n = 9 cells; pooled data at the holding voltages from −60 to −20 mV). Therefore, 90% channel closing would take place within <6 ms, enabling single spike suppression in neurons firing up to at least 180 Hz.

Our expansion of the family of natural ACRs to include 20 functional members provides the foundation for further deciphering anion conductance mechanisms of these proteins and for their molecular engineering to better suit the needs of inhibitory optogenetics.

## Methods

### Search strategy for transcriptomes

Over 2000 transcriptomes from two sequencing projects, the MMETS project^17^ and 1KP project^18^ were searched using probabilistic inference methods based on profile hidden Markov models (profile HMMs) implemented in HMMER software (version 3.1b2; ref. 35). Initially a profile HMM was built from previously known ACR sequences using default parameters. When resultant sequences from preliminary transcriptome searches were confirmed by electrophysiological measurements to be functional ACRs, these sequences were used in a recursive manner for the construction of profile HMMs to search the data again. A sample profile HMM is included in the Supplementary Material and can be viewed on Skyline web server^36^. Calling HMMER3 tools, parsing the results, and retrieving and renaming sequences was automated with Python 2.7 and the Biopython module^37^. HMMER3 tools were called with default parameters as shown in the Supplementary Note. Sequences exceeding a score of 70 were aligned to the search profile by the hmmalign tool of HMMER3. The alignment obtained was checked manually to remove partial sequences as well as almost identical sequences from the same transcriptomes that varied only in terminal positions, not in the transmembrane region. The manual editing was done with AliView 1.17.1^38^ and SeaView 1:4.5.1-1^39^. For automatic alignments MAFFT 7.164b was used^40^.

### Molecular biology

DNA polynucleotides encoding the 7TM domains of transcripts showing homology to *G. theta* ACRs optimized for human codon usage were synthesized (Genewiz, South Plainfield, NJ) and cloned into the mammalian expression vector pcDNA3.1 (Life Technologies, Grand Island, NY) in frame with an EYFP tag for expression in HEK293 cells. The sequence information was deposited in GenBank (for accession numbers see Table 1). For expression in neurons, ZipACR-EYFP fusion construct was transferred to the pFUGW vector backbone^41^. Mutants were generated using Quikchange XL kit (Agilent Technologies, Santa Clara, CA) and verified by sequencing.

### HEK293 recording

HEK293 (human embryonic kidney) cells were transfected using the ScreenFectA transfection reagent (Waco Chemicals USA, Richmond, VA). All-*trans*-retinal (Sigma) was added as a stock solution in ethanol at the final concentration of 5 μM. Measurements were performed 48–72 h after transfection with an Axopatch 200B amplifier (Molecular Devices, Union City, CA) using the 2 kHz low-pass Bessel filter. The signals were digitized at 5 kHz with a Digidata 1440 A using pClamp 10 software (both from Molecular Devices). Patch pipettes with resistances of 2–5 MΩ were fabricated from borosilicate glass. The pipette solution contained (in mM): KCl 126, MgCl_2_ 2, CaCl_2_ 0.5, Na-EGTA 5, HEPES 25, pH 7.4. The standard bath solution contained (in mM): NaCl 150, CaCl_2_ 1.8, MgCl_2_ 1, glucose 5, HEPES 10, pH 7.4. A 4 M KCl bridge was used in all experiments, and possible diffusion of Cl^−^ from the bridge to the bath was minimized by frequent replacement of the bath solution with fresh buffer. Series resistance was periodically checked during recording, and cells showing >20% change were not included in the analysis. All current-voltage dependencies were corrected for liquid junction potentials calculated using the ClampEx built-in LJP calculator. Continuous light pulses were provided by a Polychrome IV light source (T.I.L.L. Photonics GMBH, Grafelfing, Germany) at the half-bandwidth 15 nm in combination with a mechanical shutter (Uniblitz Model LS6, Vincent Associates, Rochester, NY; half-opening time 0.5 ms). The light intensity was attenuated with neutral density filters. Maximal quantum density at the focal plane of the 40× objective lens was 7.6 mW/mm^2^ for 520 nm. For measurements of the action spectra cells were illuminated with monochromatic (half-bandwidth 10 nm) of low light intensity pulses within the linear range of the dependence. The initial slope of the photocurrent was assessed from the mean amplitude of the signal recorded during the close to linear rise of the current, usually during the first 5–15 ms depending on the rate of photocurrent saturation. To avoid possible changes during the measurements, the spectral sensitivity was scanned first from the shortest wavelength to the longest one and then again in the reversed order. The data were corrected for quantum density measured for each wavelength with a calibrated photodiode. All measurements were carried out at room temperature (25 °C).

### Neuronal lentiviral transduction and recording

Lentivirus was produced in HEK293FT cells (Invitrogen) through triple-transfection of plasmids pCMV-VSVG, pΔ8.9 and pFUGW-ZipACR-EYFP. The virus was purified from the cell culture supernatant by ultracentrifugation through a 20% Sucrose layer at 20,000 rpm in an SW32 rotor for 2 hours. Neurons isolated from E18 mouse hippocampi purchased from BrainBits (Springfield, IL) by papain digestion were cultured on top of a glia feeder layer in NbActiv4 medium (BrainBits) supplemented with all-*trans* retinal (0.4 μM). BrainBits collects the brains using procedures that are approved by their Institutional Animal Use and Care Committee. Neurons were infected with lentivirus one day after plating. Patch clamp measurements were carried out 9–14 days after lentivirus infection. The same photoexcitation source and measuring setup was used as described above for HEK cells, except that neurons were bathed in Tyrode’s solution (in mM: KCl 2, NaCl 125, MgCl_2_ 1, CaCl_2_ 3, glucose 30, HEPES 25, pH 7.3) and the pipette solution contained (in mM): K gluconate 135, MgCl_2_ 2, HEPES 20, pH 7.2. Spiking was measured in the current clamp mode at room temperature (25 °C). For measurement of the spike thresholds, neurons were injected with a current ramp (0–1000 pA, 1 s) in the dark.

### Analysis of neuronal morphology

Hippocampal neurons at day 14 post-transduction were fixed in 4% paraformaldehyde in PBS, washed, permeabilized (0.3% Triton X-100 in PBS), washed once more, and blocked with 2% goat serum in PBS for 30 min. They were incubated with rabbit anti-GFP IgG (Life technologies, 1:200) and mouse anti-MAP2 IgG1 (Synaptic Systems, 1:500); or with polyclonal rabbit anti-synapsin antibody^42^ (E028, 1:1000) and guinea pig anti-Tau IgG2a (Synaptic Systems, 1:500) at 4 °C overnight. They were washed and secondarily stained with Alexa Fluor 488-conjugated goat anti-rabbit IgG and Alexa Fluor 568-conjugated goat anti-mouse IgG or Alexa Fluor 647-conjugated goat anti-guinea pig IgG at room temperature for 1 hour, washed and mounted with ProLongDiamondAntifade with DAPI mounting medium (Molecular Probes). Images were obtained with the Zen software using an LSM 510 META laser scanning microscope (Carl Zeiss, Germany). The laser wavelengths were 488 nm (KrAr), 543 nm (HeNe), and 633 nm (HeNe). Emission filters BP 490-510, BP 560-615 and LP 650 were used, respectively. Primary dendrites from randomly selected neurons were identified as MAP2-positive branches originating from the soma and crossing the circumference of a 25 μm radius. Synapses (identified by synapsin staining) were quantified using PunctaAnalyzer plugin for ImageJ in a circular area with a radius of 25 μm from the soma as described^43^.

### Statistics

Statistical data are shown as mean ± sem, unless otherwise indicated. The n values indicate the number of independent experiments (different cells, unless otherwise stated). For analysis of significance a Mann-Whitney test was used, and p values > 0.05 were considered not significant.

## Additional Information

**How to cite this article**: Govorunova, E. G. *et al*. The Expanding Family of Natural Anion Channelrhodopsins Reveals Large Variations in Kinetics, Conductance, and Spectral Sensitivity. *Sci. Rep.* **7**, 43358; doi: 10.1038/srep43358 (2017).

**Publisher's note:** Springer Nature remains neutral with regard to jurisdictional claims in published maps and institutional affiliations.

## Supplementary Material

Supplementary Material

Supplementary Dataset

## Figures and Tables

**Table 1:** GenBank accession numbers, source organisms, transcript names, action spectra maxima and protein name abbreviations of ACR homologs tested in this study.

#	Accession	Organism	Transcript name	Spectral max. (nm)	Protein name abbreviation
1	KX879674	*Geminigera cryophila* (CCMP2564)	CAMNT 0021181457*	495	*Gc*ACR_457
2	KX879675		CAMNT 0021207145*	485	*Gc*ACR_145
3	KX879676		CAMNT 0021218439*	515	*Gc*ACR_439
4	KX879677	*Geminigera* sp. (Caron Lab Isolate)	CAMNT 0013945203*	475	*G1*ACR_203
5	KX879678		CAMNT 0013979243*	N/A	*G1*ACR*_*243
6	KP171708	*Guillardia theta* (CCMP2712)	known previously^6^	515	*Gt*ACR1
7	KP171709		known previously^6^	470	*Gt*ACR2
8	KF992074	*Proteomonas sulcata* (CCMP704)	known previously^7^	520	*Psu*ACR1
9	KX879679		CAMNT 0026648973*	515	*Psu* ACR_973 (ZipACR)
10	KX879680		CAMNT 0026650433*	525	*Psu*ACR_433
11	KX879681		IRZA-2061003#	N/A	*Psu*ACR_003
12	KX879682	*Rhodomonas lens* (RHODO)	CAMNT 0019228477*	445	*Rl*ACR_477
13	KX879683	*Rhodomonas salina* (CCMP1319)	CAMNT 0012794995*	515	*Rs*ACR_995
14	KX879684	*Rhodomonas* sp. (CCMP768)	CAMNT 0042060367*	515	*R1*ACR_367
15	KX879685		CAMNT 0042061877*	520	*R1*ACR_877
16	KX879686		CAMNT 0042066447*	515	*R1*ACR_447
17	KX879687		CAMNT 0049477741*	515	*R1*ACR_741
18	KX879688		CAMNT 0049533799*	520	*R1*ACR_799
19	KX879689	*Rhodomonas* sp. (CCAC1630)	IAYV-2004853#	515	*R2*ACR_853
20	KX879690		IAYV-2007142#	490	*R2*ACR_142
21	KX879691	not classified (CCMP2293)	CAMNT 0022112887*	535	*C1*ACR_887
22	KX879692		CAMNT 0022176023*	445	*C1*ACR_023

Three previously known ACRs are also included.

^*^Transcripts from the MMETS project.

^#^Transcripts from the 1 KP project.
