# Supplementary material for: The Expanding Family of Natural Anion Channelrhodopsins Reveals Large Variations in Kinetics, Conductance, and Spectral Sensitivity
Source: Sci Rep. 2017 Mar 3;7:43358. doi: 10.1038/srep43358 (PMC5335703; doi:10.1038/srep43358)
Supplement: Supplementary Material [file srep43358-s1.pdf]

Supplementary Material

The Expanding Family of Natural Anion Channelrhodopsins Reveals Large Variations in Kinetics, Conductance, and Spectral Sensitivity

Elena G. Govorunova[1], Oleg A. Sineshchekov[1], Elsa M. Rodarte[2], Roger Janz[2], Olivier Morelle[3], Michael Melkonian[3], Gane K.-S. Wong[4,5], and John L. Spudich[1*]

[1]Center for Membrane Biology, Department of Biochemistry & Molecular Biology, The University of Texas Health Science Center at Houston, McGovern Medical School, Houston, Texas, USA; [2]Department of Neurobiology & Anatomy, The University of Texas Health Science Center at Houston, McGovern Medical School, Houston, Texas, USA; [3]Institute of Botany, Cologne Biocenter, University of Cologne, Cologne, Germany; [4]Departments of Biological Sciences and of Medicine, University of Alberta, Edmonton, Alberta, Canada; [5]BGI-Shenzhen, Shenzhen, China

[*]Correspondence should be addressed to J.L.S. (John.L.Spudich@uth.tmc.edu).

Supplementary Note

Calling tools from HMMER3:

For the construction of profile HMMs the tool "hmmbuild" was used without additional parameters where "hmmfile" is the name of the produced profile and "msafile" is the name of the used multiple sequence alignment file:

hmmbuild hmmfile msafile

The profile search was performed using the tool "nhmmer". The used parameters only affect the computation time, the file naming and the line width of the output.

nhmmer --cpu 3 -o hmmout -A hmmalignment --tblout tableresults --notextw hmmfile transcriptomefile

Found sequences were aligned to the profile using the tool "hmmalign":

hmmalign hmmfile seqfile

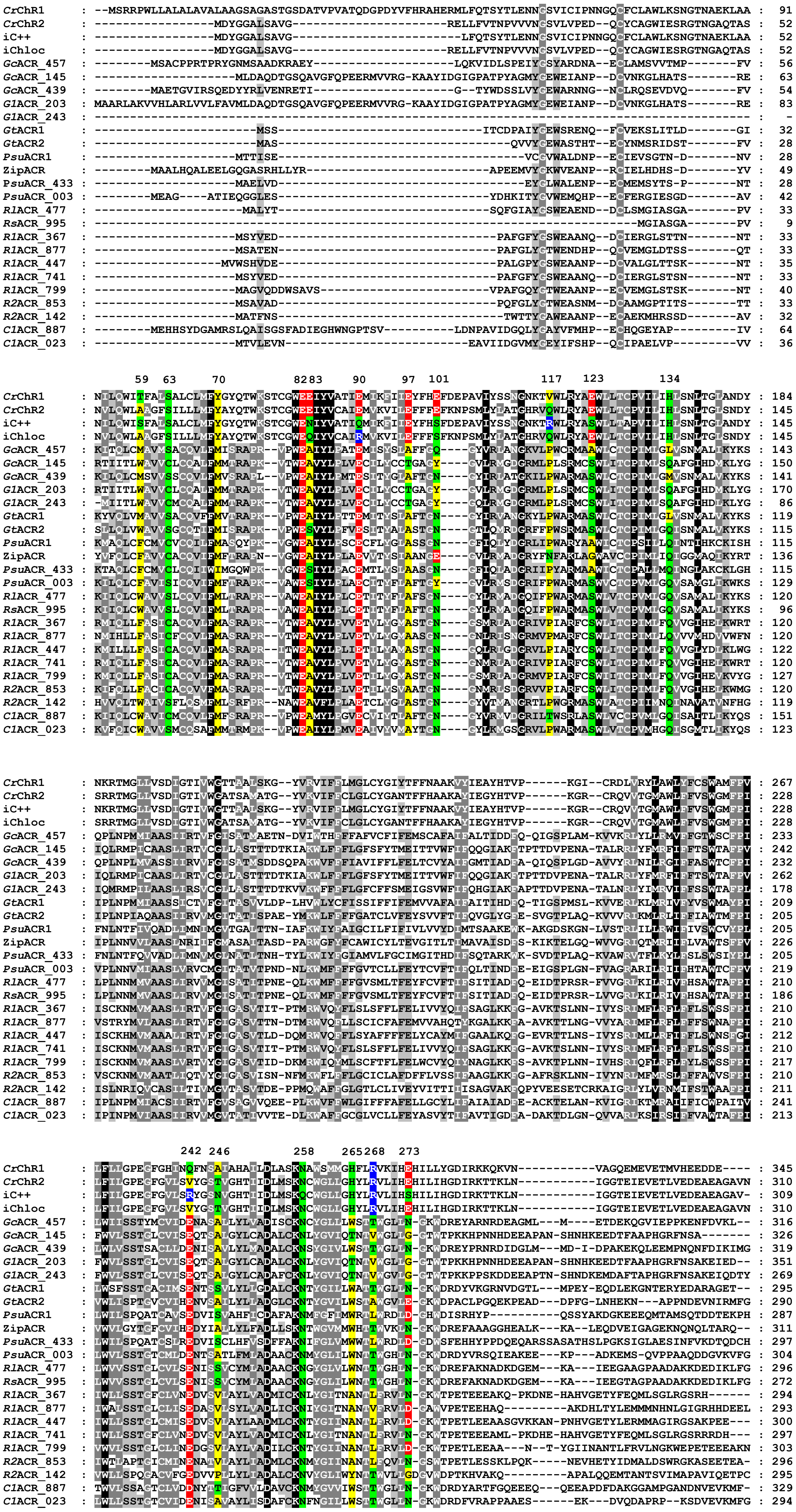

59 63 70 82 83 90 97 101 117 123 134
242 246 258 265 268 273

Supplementary Figure S1. ClustalW protein alignment of seven-helix transmembrane domains of ACR homologs tested in this study. Also included are two CCRs (lines 1 & 2), two engineered Cl^--conducting CCR mutants (lines 3 & 4) and three previously characterized ACRs (lines 10-12). On the right are shown the numbers of the last residue in each line. The residue numbers on top of the alignment correspond to the sequence of *Cr*ChR2. The red lines show transmembrane helices according to 3ug9 structure.

The four levels of gray shading reflect the degree of conservation (from the darkest to the lightest): 100% conserved, ≥80% conserved, 60%-80% conserved, and <60% conserved. Colored residues are positions in which mutations have been introduced to confer Cl^- selectivity to CCRs mutants and those corresponding to the residues that contribute to the central and inner gates in the high-resolution crystal structure 3ug9 of a hybrid CCR. The color code is: blue, positively charged residues; red, negatively charged residues; green, polar residues; yellow, non-polar residues.

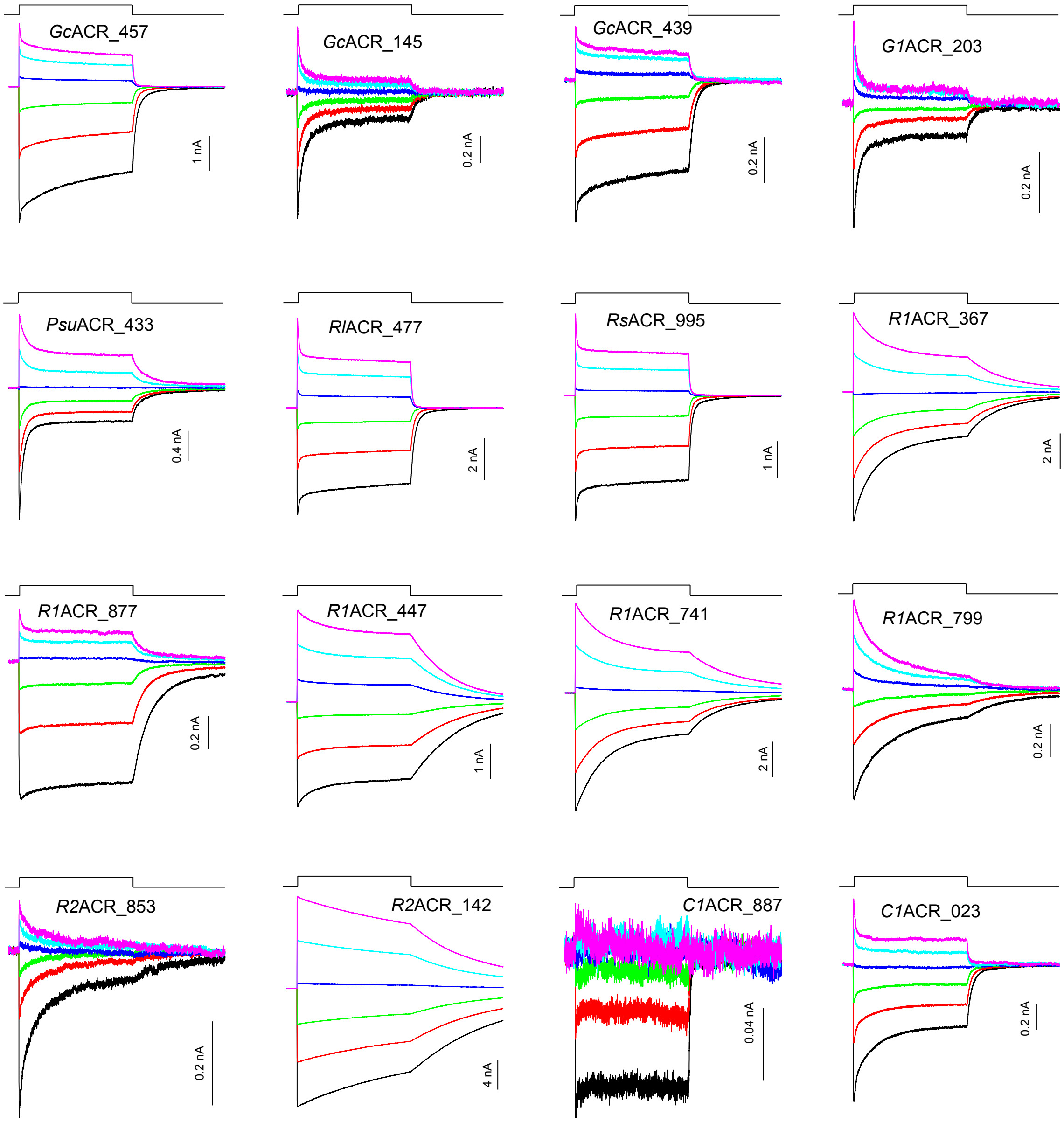

Supplementary Figure S2. Representative series of photocurrents generated by ACR homologs expressed in HEK293 cells. The traces were recorded in the standard bath in response to a 1-s light pulse (shown on top; also serves as a time scale bar). Note the different scaling of the Y axis in different panels. The holding voltage was changed in 20-mV steps from -60 mV at the amplifier output (bottom trace). Abbreviated protein names are shown in each panel (see Table 1 in the main text for their accession numbers and source organisms).

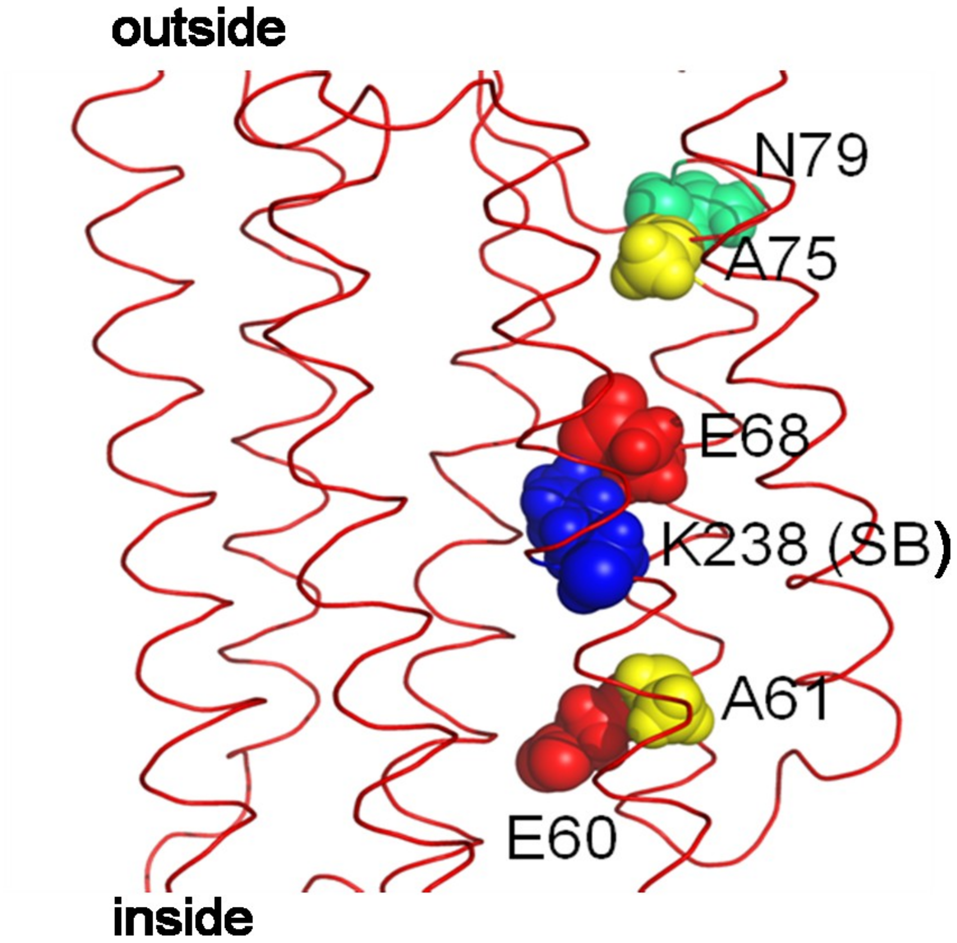

Supplementary Figure S3. **A homology model of *Gt*ACR1 built on Robetta server using the 3ug9 template**. Shown are the side chains of the residues that were replaced with glutamates for testing in this study (Ala61, Ala75 and Asn79), conserved glutamates in helix 2 (Glu60 and Glu68) and the Schiff base (Lys238).

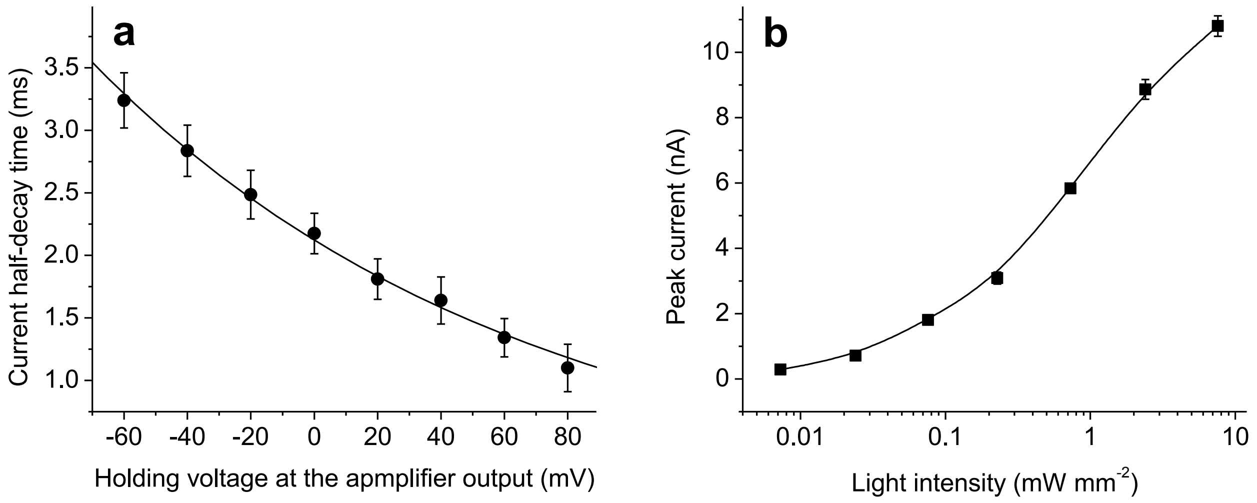

Supplementary Figure S4. Properties of ZipACR photocurrents in HEK293 cells. (**a**) The dependence of ZipACR photocurrent half-decay time on the holding voltage. The data points are the mean values ± sem (n = 6 cells). (**b**) The dependence of ZipACR photocurrent amplitude on the stimulus intensity. The data points are the mean values ± sem (n = 3 cells).

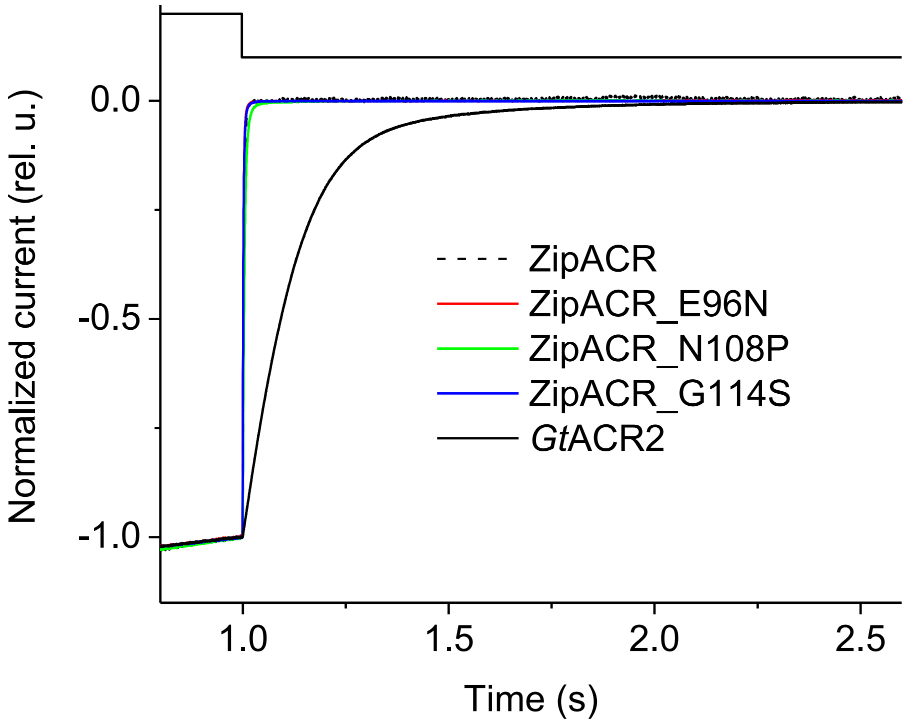

Supplementary Figure S5. Photocurrent decay rate in ZipACR is not affected by the E96N, N108P and G114S mutations. The currents traces were measured in HEK293 cells in standard buffer at -60 mV and normalized at the end of a 1-s light pulse the time course of which is schematically shown on top. A trace recorded from *Gt*ACR2 is shown for comparison.

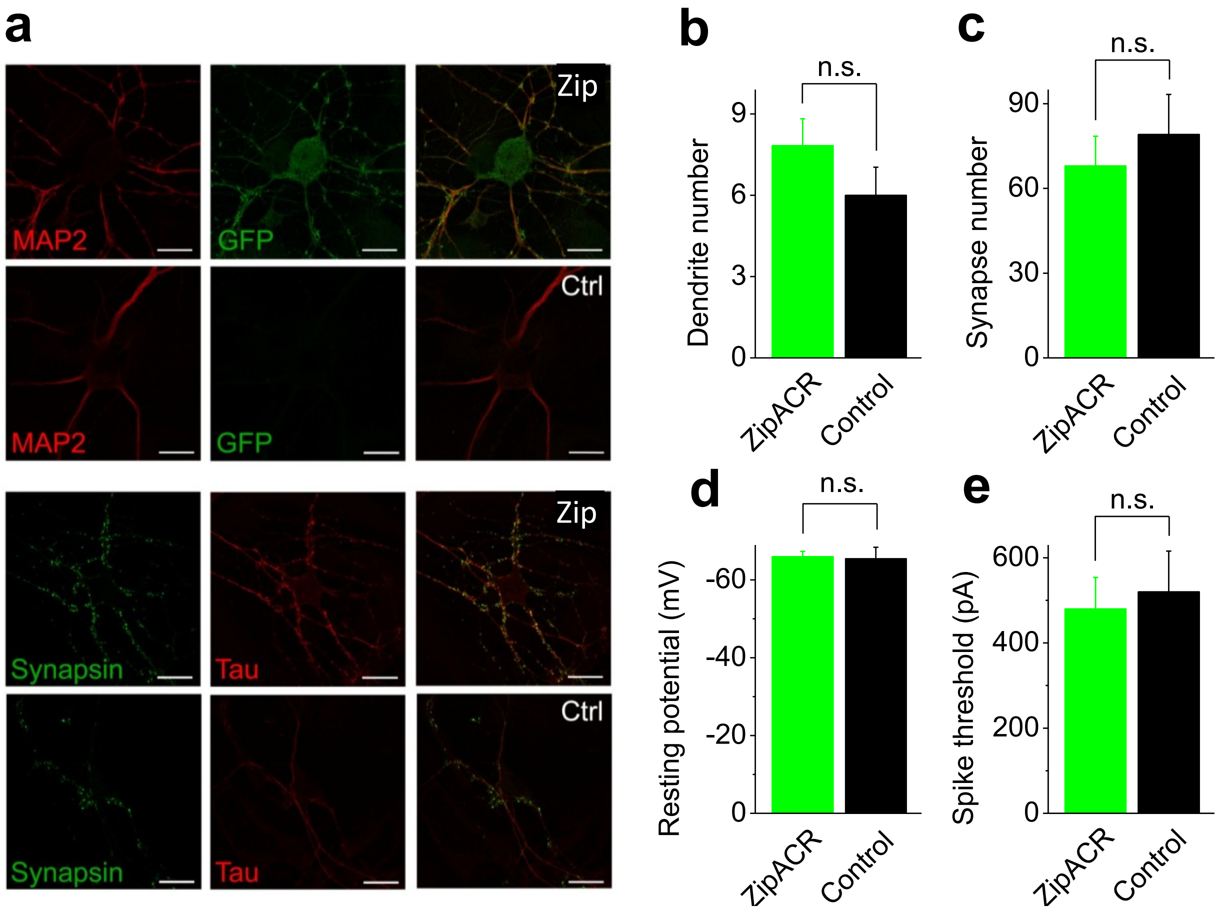

Supplementary Figure S6. ZipACR expression does not affect neuronal morphology and physiology. (**a**) Representative images of cultured mouse hippocampal neurons expressing ZipACR (Zip) or control non-transduced neurons (Ctrl). The neurons were stained with antibodies against MAP2 (a dendritic marker), GFP, Synapsin (a synaptic marker) and Tau (an axonal marker). Scale bar 20 µm. (**b**) The number of primary dendrites per neuron at a 25 µm radius around the center of the soma. (**c**) The number of synapses per neuron in a 25 µm circle around the center of the soma. (**d**) The resting membrane potential in the dark. (**e**) The spike threshold in the dark (the injected current at the time of first spike induced by ramp stimulation). The data in panels b-c are the mean values ± sem (n = 5-12 cells). There was no significant difference between transduced and non-transduced neurons (p values by Mann-Whitney test were >0.05).

Supplementary Table S1. Cryptophyte transcriptomes searched in this study for ACR homologs.

	Project code	Organism	Habitat	Search output
1.	MMETSP0038	*Cryptomonas paramaecium*	freshwater	No ACR homologs identified, low quality
2.	MMETSP0043	*Hemiselmis andersenii*	marine	ACR homologs identified
3.	MMETSP0046	*Guillardia theta*	marine	ACR homologs identified & confirmed previously [9]
4.	MMETSP0047	*Chroomonas cf. mesostigmatica*	marine	No ACR homologs identified, low quality
5.	MMETSP0484	*Rhodomonas lens*	ubiquitous	ACR homologs identified & confirmed
6.	MMETSP0799	*Geminigera cryophila*	marine	ACR homologs identified & confirmed
7.	MMETSP0986	Unidentified eukaryote sp.	marine	ACR homologs identified & confirmed
8.	MMETSP0987	Unidentified eukaryote sp.	marine	ACR homologs identified & confirmed
9.	MMETSP0988	Unidentified eukaryote sp.	marine	ACR homologs identified
10.	MMETSP0989	Unidentified eukaryote sp.	marine	ACR homologs identified
11.	MMETSP1041	*Hemiselmis andersenii*	marine	ACR homologs identified
12.	MMETSP1042	*Hemiselmis andersenii*	marine	ACR homologs identified
13.	MMETSP1043	*Hemiselmis andersenii*	marine	ACR homologs identified
14.	MMETSP1047	*Rhodomonas salina*	marine	ACR homologs identified & confirmed
15.	MMETSP1048	*Hanusia phi*	marine	ACR homologs identified
16.	MMETSP1049	*Proteomonas sulcata*	marine	ACR homologs identified & confirmed previously [12-13]
17.	MMETSP1050	*Cryptomonas curvata*	freshwater	No ACR homologs identified
18.	MMETSP1091	*Rhodomonas* sp.	marine	ACR homologs identified & confirmed
19.	MMETSP1101	*Rhodomonas abbreviata*	marine	ACR homologs identified
20.	MMETSP1102	*Geminigera* sp.	marine	ACR homologs identified & confirmed
21.	MMETSP1355	*Hemiselmis tepida*	marine	No ACR homologs identified
22.	MMETSP1356	*Hemiselmis virescens*	marine	ACR homologs identified
23.	MMETSP1357	*Hemiselmis rufescens*	marine	ACR homologs identified
24.	MMETSP1389	*Rhodomonas* sp.	marine	ACR homologs identified & confirmed

25.	BAKF	*Cryptomonas curvata*	freshwater	No ACR homologs identified, low quality
26.	IAYV	*Rhodomonas* sp.	marine	ACR homologs identified & confirmed
27.	IRZA	*Proteomonas sulcata*	marine	ACR homologs identified & confirmed previously [12-13]
28.	MJMQ	*Hemiselmis virescens*	marine	No ACR homologs identified
29.	ROZZ	*Chroomonas* sp.	freshwater	No ACR homologs identified

Supplementary Table S2. Numerical data on ACR diversity for Fig. 4 in the main text. The mean and sem values of the peak current, inactivation (measured as the difference between the peak value and the value at the end of the 1-s light pulse divided by the peak value, per cent), half-decay time (the time till the current reached 50% amplitude after the light-off) and the number of sampled cells for each ACR homolog tested in this study. The data for the previously characterized *Gt*ACR1, *Gt*ACR2 and *Psu*ACR1 are also included for comparison.

#	Protein name abbreviation	Mean peak current (nA)	Sem peak current (nA)	Mean inactivation (%)	Sem inactivation (%)	Mean half-decay time (ms)	Sem half-decay time (ms)	Number of cells
1	*Gc*ACR_457	4.8	0.4	41.7	1.3	20.4	1.3	5
2	*Gc*ACR_145	1.2	0.3	82.9	0.8	25.0	1.0	3
3	*Gc*ACR_439	1.2	0.5	31.7	3.8	37.7	7.9	3
4	*G1*ACR_203	0.8	0.4	76.1	3.0	38.0	2.5	3
5	*G1*ACR_243							
6	*Gt*ACR1	7.5	0.9	12.0	1.2	128.4	15.8	12
7	*Gt*ACR2	7.4	1.0	20.7	1.6	75.7	8.1	8
8	*Psu*ACR1	0.7	0.2	43.8	1.6	21.3	0.9	16
9	*Psu*ACR_973 /ZipACR	10.7	1.3	17.0	2.3	3.2	0.2	6
10	*Psu*ACR_433	1.2	0.1	79.5	2.2	26.0	1.5	3
11	*Psu*ACR_003							
12	*Rl*ACR_477	5.0	0.5	27.7	1.7	29.7	3.2	7
13	*Rs*ACR_995	3.9	1.1	32.2	3.9	14.7	3.2	3
14	*R1*ACR_367	6.0	0.4	74.0	0.5	145.5	38.5	3
15	*R1*ACR_877	1.0	0.1	12.5	1.1	115.0	4.0	3
16	*R1*ACR_447	2.3	0.5	34.1	7.2	182.3	67.1	3

17	*R1*ACR_741	8.8	0.9	73.6	1.3	160.4	11.7	5
18	*R1*ACR_799	0.6	0.6	73.8	5.0	257.5	15.5	3
19	*R2*ACR_853	0.2	0.1	83.6	1.4	233.0	83.0	4
20	*R2*ACR_142	15.0	1.6	43.5	2.7	305.8	36.0	12
21	*C1*ACR_887	0.05	0.04	27.5	9.3	8.0	2.5	3
22	*C1*ACR_023	0.6	0.5	51.5	8.9	38.7	9.8	3
